# Assessment of Productivity and Quality of Publications Derived From Doctoral Theses and the Scientific Performance of Graduates at a Romanian Medical and Pharmacy University

**DOI:** 10.7759/cureus.86947

**Published:** 2025-06-29

**Authors:** Andreea I Pop, Mira Florea, Simona Mirel, Lucia M Lotrean

**Affiliations:** 1 Department of Community Medicine, Research Center in Preventive Medicine, Health Promotion and Sustainable Development, University of Medicine and Pharmacy Iuliu Hatieganu, Cluj-Napoca, ROU; 2 Department of Community Medicine, Discipline of Family Medicine, Iuliu Hatieganu University of Medicine and Pharmacy, Cluj-Napoca, ROU; 3 Department Fourth Pharmacy, Discipline of Medical Devices, Iuliu Hatieganu University of Medicine and Pharmacy, Cluj-Napoca, ROU; 4 Department of Community Medicine, Research Center in Preventive Medicine, Health Promotion and Sustainable Development, Iuliu Hatieganu University of Medicine and Pharmacy, Cluj-Napoca, ROU

**Keywords:** doctoral student, medical education and research, publication rate, scientific publication indicators, thesis

## Abstract

Objectives

This study focuses on assessing the productivity and quality of publications resulting from doctoral research as well as the scientific performance of individual researchers at the University of Medicine and Pharmacy in Cluj-Napoca, Romania. It analyzes scientific publication rates before, during, and after doctoral school. We analyzed the bibliometric indicators of publications indexed in the Web of Science database, which are derived from doctoral research and feature graduates as first authors, as well as the factors that might impact the research outcomes.

Design and setting

This investigation is a longitudinal observational study conducted from January to March 2023 at Iuliu Hatieganu University of Medicine and Pharmacy in Cluj-Napoca, Romania. The study employed both descriptive and analytical research methods. The sample comprises public information about 169 doctoral (PhD) candidates who successfully completed the doctoral program between 2019 and 2021. We collected data from various public databases, including the university's website, the university library, and the Web of Science Core Collections.

Main outcome measures and research productivity

The primary outcome measures include gender, graduation years, qualification, teaching position, doctorate final grade, and Hirsch index (H-index). The productivity of research includes the total number of WoS-indexed publications, conference proceedings, and manuscripts in various databases. Research quality and visibility are represented by total citations, journal impact factors and journal rankings. Factors influencing research dissemination: The factors that influence research dissemination include the number of co-authors, financial support, and interdisciplinary/international collaborations. The publication timeline includes research output before, during, and after graduation.

Results

At the University of Medicine and Pharmacy Iuliu Hatieganu, doctoral theses produced high-quality research; 332 (91%) of the publications were indexed in WoS journals with JIF, 136 (38%) of these publications were in top-ranked journals, and 298 (81%) of the publications received citations. Before they graduated, graduates also published the findings of other studies, and some of them continued publishing after graduation. The graduation qualification strongly correlated with the graduates' publications' productivity, impact, and visibility. The study found that financial support and international collaboration had a positive influence on the publication's visibility and quantity.

Conclusions

This study provides new quantitative and qualitative evidence that can enhance discussions regarding the assessment of academic performance and the mentoring of doctoral students. Our study identifies various factors that can influence the results of research.

## Introduction

Globally, medical professionals demonstrate a strong interest in pursuing doctoral studies, the highest level of academic education, enabling the attainment of a level 8 certification in accordance with the European Qualifications Framework (EQF)/Common European Framework of Reference for Languages (CEFR) and the National Qualifications Framework [1]. Despite not being necessary for practicing medicine, professionals increasingly view doctoral studies as "an almost mandatory final degree," a stage of qualification that is not optional in academic medicine [2]. According to Romanian education legislation, the minimum academic rank of lecturer in a university requires a PhD degree or enrollment in a doctoral program [3].

Regarding the benefits of conducting doctoral studies, there are two principal outputs: one intended for the graduate and the other for the broader community. Thus, doctoral studies play a crucial role in contemporary higher education by enhancing research and cultivating highly qualified experts who enhance both academic and scientific fields. Previous studies showed that candidates' completion of a thesis can contribute to their personal development. The cultivation of specialized abilities to gain expertise, the capacity to function in a research team, and enhanced communication skills during the dissemination of research outcomes, as well as the development of academic identity, are all important aspects to consider. Those who have successfully completed a doctoral program tend to perceive their academic competence as significantly higher than those who have not [2,4,5]. However, other studies suggest that young scientists and new recruits have produced considerable knowledge and contributed significantly to various important scientific innovations [6]. The dissemination of research findings via scientific publications and conferences can enhance the visibility of affiliated universities [7]. In many countries, governmental and other funding organizations utilize research productivity and quality measures (e.g., publications, citations, and journal impact factors) as primary criteria for distributing financial resources. The primary justification for supporting incentives is that allocating financial rewards to top performers is likely to provide superior outcomes and encourage better results [8].

For scientific research to exert impact, effective communication of its findings is imperative. The most favored approach for disseminating doctoral research is through the publication of results in scientific journals that have undergone a rigorous peer-review process. The publication process that involves peer review can serve as evidence of the significance of research. In this light, the assessment of research quality is often based on publication in journals [9]. Nevertheless, publication rates serve as a metric for evaluating both individual and institutional performance. Universities thus mandate a minimum number of publications for each doctoral candidate. Due to this reality, Doctor of Philosophy (PhD) students face significant challenges in increasing their publication output and maximizing their visibility [4,7].

The relation between publishing quality and productivity has received increased attention, as the primary motivation for publishing has shifted towards securing promotions, achieving high scientific rankings, and enhancing job security. The intense pressure to produce and disseminate research findings has resulted in a proliferation of insignificant studies, with a high incidence of plagiarism, contributing to a rise in the rate of retractions [10-13]. According to Giesler et al., given the consistently high number of doctorates awarded, it is appropriate to assume that it is impossible to satisfy the requirement that every doctorate must, primarily, contribute to a qualitative or recognizable improvement in science [2,14].

A group of statistical and mathematical indices, defined as bibliometric indicators, is used to assess the quality and quantity of publications. Bibliometric indicators include various metrics, such as the quantity of papers produced, the frequency of article citations, the number of co-authors, the number of publications featured in the top 10%, the Hirsch index (H-index), the position index, co-publications, the number of papers per researcher, and the journal impact factor (JIF) [15].

Among the numerous bibliometric indices utilized for evaluating an individual researcher's performance, the H-index and JIF are the most popular and frequently utilized [16]. Based on the quantity and quality of an author's publications, the H-index evaluates their overall academic impact. The H-index is a metric that integrates a researcher's quantitative (productivity) and qualitative (citations) research activity into a singular numerical value. This implies that achieving an elevated H-index requires more than just a few highly cited papers or an excessive number of papers with minimal citations [17]. A prominent metric for evaluating research quality, JIF serves as a measure of a journal's significance within its field of expertise. It measures the frequency of citations for the "average article" in a journal over a two-year period. More citations boost the journal's prestige and impact factor [18,19].

Furthermore, researchers have developed numerous methodologies to evaluate the scientific output of individual researchers, including the cumulative impact factors of the journals (CJIF) in which their publications appear. This technique assigns an index impact to the researcher, not based on the papers themselves, but from the journals in which they published manuscripts [20].

Academics and the scientific community recognize Clarivate as the organization responsible for calculating the impact factor. The Web of Science Core Collection provides access to Clarivate's annual Journal Citation Reports (JCR), an integrated publication that provides detailed information on academic journals across a wide range of academic disciplines. JCR, initially published as part of the Science Citation Index, has evolved into a distinct service that integrates citation data from the Science Citation Index Expanded (SCIE) and Social Sciences Citation Index (SSCI) databases. The 2023 edition includes journals from the Arts and Humanities Citation Index (AHCI) and the Emerging Sources Citation Index (ESCI) [21]. (SCIE, SSCI, AHCI, and ESCI are key citation databases within the Web of Science Core Collection, which also includes the Book Citation Index (BCI) and the Conference Proceedings Citation Index (CPCI) [22]. Globally, people recognize this database as the oldest, most extensively utilized, and highly regarded source of scientific publications and citation data [23]. In Romania, the performance indicators from the Web of Science (WoS) database are used to assess the minimum requirements for completing a doctoral thesis successfully and advancing within the academic field [24].

All medical and pharmacy universities in Romania want to improve their research output to align with international standards. To our knowledge, Romanian universities have not conducted any recent studies focusing on high-quality publications derived from doctoral research to evaluate the knowledge production of doctoral graduates. This study specifically focuses on the quantity and quality of scientific publications derived from doctoral research, the continuation of research after graduation, and evaluating several factors that can influence research results. Thus, this study assesses performance metrics associated with the dissemination of doctoral research findings. We also monitored other aspects, including productivity indicators for non-doctoral research articles published before, during, and after the doctoral program's completion. Furthermore, socio-demographic aspects were examined.

Several international studies on literature have focused on analyzing the productivity of developing these publications among graduates of PhD programs [2,7,11,25-27]. There has been limited research to evaluate the quality of publications from medical doctoral studies [28]. This research holds significant value in providing an overview of the factors that contribute to enhancing the productivity and visibility of candidates and their publications, particularly in the current context where there is a growing demand for PhD students to publish during their candidacies. Also, the findings aim to provide actionable insights for improving the effectiveness of doctoral programs and aligning them with international standards.

The current research aimed to use bibliometric indicators to evaluate the publication rates and characteristics of scientific manuscripts related to medical doctoral theses defended between 2019 and 2021, as well as the academic and socio-demographic characteristics of the graduates. This study intends to provide a more precise focus on the following research objectives. The identification of socio-demographic indices includes gender, year of faculty graduation, and year of doctoral school graduation. We evaluate candidates' academic performance using the cumulative impact factor, the H-index, their final doctorate grade, and their placement within the academic environment. We evaluate the knowledge output of doctoral research by looking at the total number of publications, specifically those indexed by WoS. We use the impact factor (JIF), the number of citations in Web of Science, and journal ranking (per Journal Citation Reports) to evaluate the visibility of our articles. We are identifying several factors that could potentially contribute to the publication of research findings from doctoral studies. The factors under consideration are the number of co-authors, research funding, the extent of interdisciplinary collaboration, and the level of international collaboration. We evaluate publication trends by considering results published outside of doctoral research.

## Materials and methods

The present investigation constitutes a component of doctoral research based on evaluating the participation of medical students and young physicians in research and volunteerism activities. This investigation is a longitudinal observational study conducted from January to March 2023 among PhD graduates. The study employed both descriptive and analytical research methods, which assessed socio-demographic factors and bibliometric indices, as well as various aspects of the type of collaboration and funding of studies.

Study sample and procedure for data collection

The sample comprises public information about 169 PhD candidates who successfully completed the doctoral program at the University of Medicine and Pharmacy in Cluj-Napoca, earning the academic distinction of Doctor of Medical Sciences between 2019 and 2021. Thus, the study included 49 (29%) graduates from 2019, 60 (35.5%) from 2020, and 60 (35.5%) from 2021. Out of the total number, 59 individuals (35%) were identified as males, and 110 individuals (65%) were identified as females.

We used a variety of public databases to gather study data. Firstly, we accessed the university's website, where candidates publish their CVs immediately before their thesis presentation. Secondly, we obtained the full texts of these from the university's library resources. Finally, we consulted Web of Science (WoS) and Journal Citation Reports (JCR) to gather information about scientometric analyses. We selected this database for collecting and evaluating bibliometric and scientometric indicators due to its utilization for assessing the minimal criteria required for the completion of the doctoral thesis in medicine in Romania. Therefore, we deemed it appropriate to use bibliometric and scientometric indicators to evaluate the quality and productivity of doctoral research publications. This analysis specifically concentrated on publications found in journals with an impact factor, indexed in the SCIE and SSCI databases of the WoS. Additionally, we conducted an analysis of proceedings papers without impact factors using the WoS database. This organization is well known for producing high-quality publications. The Iuliu Hatieganu University Library provided us access to the Web of Science Core Collection platform through its subscription.

We analyzed the socio-demographic indices by scrutinizing the complete lists of graduates posted on the institution's website, along with their CVs. Therefore, we considered the following variables for each applicant: gender, derived from student enrollment records; year of university graduation; year of doctoral school graduation; graduation qualification; and university teaching staff (lecturer), as indicated on their CV.

We examined the university's website for the doctorate's final grade (good, very good, excellent) and the WoS database for each candidate's H-index to assess their academic performance. We assessed the productivity of the graduates by analyzing the complete texts of their theses, which included a statement of all publications arising from their doctoral research. We quantified the total number of WoS publications, original full-text articles, systematic reviews, and meta-analyses indexed in journals with or without impact factors, for each candidate serving as the first author and indexed in the WoS database. Additionally, we separately measured the number of publications resulting from conference results, specifically, the number of WoS proceedings related to these. Our study also quantified the total number of manuscripts that were an important part of theses and published in other international databases.

To assess the quality and visibility of the articles, the variables considered were the sum of the citations (according to the Wos database), the impact factor of the journal, the classification of the journal as Q1, Q2, Q3, or Q4 rankings (via JCR) and CJIF (calculating the total JIFs of the journals in which manuscripts are published for each graduate). To determine the variables that may positively influence the dissemination of doctoral research findings, we examined the number of co-authors involved in each publication, the presence of financial support for the studies, and the existence of interdisciplinary or international partnerships. We determined the funding based on the acknowledgments in the publications and scrutinized their CVs to verify whether the funding was specifically for the graduate or not for a co-author. Doctoral students typically apply for funding through the University Iuliu Hatieganu's doctoral research program and include this information in their CV or the acknowledgements section of their publications. We identified interdisciplinary and international collaboration based on the employment or departmental affiliations derived from graduate publications. We also analyzed the Web of Science Categories and Research Areas assigned to the co-author profiles to confirm this data.

We quantified research results published pre-, during, and post-graduation in manuscripts not integrated in doctoral theses. To identify pre-graduation manuscripts, we utilized the candidates' CVs to distinguish articles unrelated to their doctoral research. We utilized the WoS database to find post-graduation manuscripts by searching for the candidate's name and university affiliation.

Data analyses

The prevalence of the issues examined was calculated for the entire population that was included in the study. Descriptive data was obtained for both the entire sample and each individual candidate. The data is reported in the form of mean values with standard deviations and range, or as counts with corresponding percentages.

The study employed linear regression analysis to evaluate the association between specific variables and the quantity of publications indexed in WoS. Additionally, we utilized linear regression to investigate the factors associated with the Hirsch index level and the cumulative impact factor for the candidates. For the regression analyses, we used the same independent variables (gender, year of university graduation, year of doctorate graduation, graduation grade, university teaching staff status, presence of at least one funded publication, presence of at least one publication with interdisciplinary collaboration, presence of at least one publication with international collaboration) for each dependent variable. However, for certain dependent variables (cumulative impact factor, number of citations, number of publications after graduation, and the H index), we included additional specific independent variables for each individual year. Subsequently, we opted for the forward method to ensure that the analysis exclusively encompassed statistically significant data (p < 0.05). Furthermore, using this method of statistical analysis was helpful in determining which factors are associated with the rate of publication among those who have recently completed their doctoral research. The statistical analysis of the data was done using the SPSS software package, version 22 (IBM Corp., Armonk, NY). The findings considered statistically significant are reported at a significance level of 0.05.

## Results

Assessment of publication productivity

As first author, the graduates published 536 manuscripts indexed in the WoS database or other international databases. The study revealed that the number of WoS publications was 366 (68% of all related publications). Additionally, they published 143 first-authored manuscripts (28% of all publications at graduation), derived from other studies, not from doctoral research, before and during their doctoral program. Thus, the authors had 509 WoS publications at the time of graduation, including both publications derived and not derived from doctoral research. Additionally, graduates continued to publish after graduation; at the time of the evaluation, we identified 154 WoS publications (Table 1).

**Table 1:** Descriptive data regarding publications productivity WoS: Web of Science.

	N (Reported at the sample)		Minimum (pubications per graduate)	Maximum (pubications per graduate)	Mean (pubications per graduate)	Std. Deviation (pubications per graduate)
Number of all these related publications (indexed in WoS database and in other international database)	536	1		9	3.17	1.323
Number of these-related publications (indexed WoS)	366	1		8	2.17	1.290
Number of these-related publications listed in another database (not WoS database)	170	0		4	1.01	0.948
Number of these unrelated publications (indexed WoS)	143	0		8	0.85	1.673
Number of all publications at graduation (related and not related with theses) published before and during PhD candidature (indexed WoS)	509	1		9	3.01	1.994
Number of publications after graduations (indexed WoS)	154	0		10	0.91	1.577

Assessment of publication quality

We assess the quality of publications based on their impact and visibility. We assessed the visibility of our participants' published articles in JCR-listed journals, which were derived from their doctoral research. The publications derived from doctoral research were quoted a total of 3122 times (Table 2). Journals with JIF accepted are 332 (91%) of WoS publications derived from doctoral research, while journals without an impact factor (IF) and accepted are 34 (9%), and WoS Proceedings published are 22 (6%). Journals with a JIF range of 1 to 3 indexed just over half of the published articles, while journals with a JIF range of 3 to 5 accepted 70 (19%) of the publications. About 24 (6%) of the publications were in high-impact journals with a JIF greater than 5, while the remaining 49 (14%) were in low-impact magazines with a JIF less than 1 (Table 2).

**Table 2:** Characteristics of the theses-related WoS publications WoS: Web of Science, PhD: Doctorate of Philosophy, JIF: Journal Impact Factor.

	(N=366)	%
Number of WoS publications accepted in journals with an impact factor	332	91
Number of WoS publications accepted in journals without an impact factor	34	9
Number of WoS proceedings publications	22	6
Publications with interdisciplinary collaboration	318	87
Publications with international collaboration	65	18
Publications supported by research grants	162	45
Co-authors (without the first author represented by a PhD student)		
<=5 Co-authors	172	47
>5->10 Co-authors	160	44
>10 Co-authors	34	9
Quartile		
Publications indexed in journals not included in Quartiles	34	9
Q1 Publication	65	18
Q2 Publication	71	20
Q3/Q4 Publication	196	54
JIF Range of journals indexing publications derived from doctoral research		
JIF <1	49	14
JIF range 1-<3	189	52
JIF range >3-<5	70	19
Publication with JIF >5	24	6
Cites		
0 cites/ publication	68	19
<10 cites/publication	191	52
10->20 cites/ publication	63	17
>20 cites/ publication	44	12
Number of all sample citations (theses-related WoS articles)	3122	

All graduates reported at least one WoS publication indexed in journals with JIF by the time of graduation, with a mean of 1.96 (SD = 1.210) WoS publications. A total of 149 (88%) reported one to three WoS publications derived from doctoral research, while the maximum number was eight publications. A number of 156 (92%) of graduates published one to three articles in journals with JIF, while 13 (8%) had four to eight publications in such journals. Additionally, we evaluated journal visibility using JCR's ranking system, which categorizes journals by research domain. We assigned a quartile rank (Q1 to Q4) to each publication derived from doctoral research in a JCR-listed journal. The distribution of published manuscripts was as follows: 65 (18%) in the first quartile, 71 (20%) in the second quartile, and 196 (54%) in the third and fourth quartiles (Table 2).

A total of 38 (22%) of the graduates had published at least one manuscript in a Q1-indexed journal. The highest number of publications per participant was seven, with a mean of 0.38 (SD = 0.92). A sum of 59 (34%) of the participants published at least one manuscript in Q2-indexed journals. The maximum number of publications per participant was two, with a mean of 0.42 (SD = 0.62). In contrast, most graduates, 132 (76%), had published at least one manuscript in journals with indexing in Q3 or Q4. The highest number of publications per graduate was five, with a mean of 1.14 (SD = 0.88). Most graduates had at least one cited publication, and the highest number of citations per candidate was 207. Candidates have a mean of 18.15 citations (SD = 32.08) (Table 3).

**Table 3:** Specific characteristics of publications derived from doctoral research WoS: Web of Science, JIF: Journal Impact Factor.

	N (Graduates with at least one publication)	%	Minimum (pubications per graduate)	Maximum (pubications per graduate)	Mean (pubications per graduate)	SD (pubications per graduate)
Supported by research grants	87	51	0	7	0.94	1.25
Interdisciplinary collaboration	160	93	0	7	1.85	1.24
International collaboration	41	24	0	5	0.38	0.79
WoS publications indexed in journals with JIF	169	100	1	8	1.96	1.210
Rate of cites	154	90	0	207	18.15	32.08
Q1 Publication	38	22	0	7	0.38	0.92
Q2 Publication	59	34	0	2	0.42	0.62
Q3/Q4 Publication	132	76	0	5	1.14	0.88

Doctoral theses publication characteristics

According to the authors, a considerable proportion of graduates, 160 (93%), have at least one publication with interdisciplinary collaborations. Furthermore, 41 (24%) of graduates have at least one publication with international collaborations. We found that 142 (47%) of publications had between one and five co-authors, while 160 (44%) had a range of six to 10 co-authors. Research grants facilitated the publication of 162 (45%) of the studies (Table 3).

Scientometric indicators

The mean cumulative JIF of publications in doctoral theses was 4.712 (SD = 5.94), with a maximum value of 48.56. The study found that the mean H-index of the graduates was 3.65 (SD 2.95), with a maximum value of 20. Also, the study's findings suggest that a substantial majority of participants obtained a grade of "very good” upon completing their studies. In their CVs, 103 (60%) of the participants reported their employment as university teaching staff (Tables 4, 5).

**Table 4:** Scientometric characteristics regarding graduates CJIF: Clarivate Journal Impact Factor, H-index: Hirsch index.

N =169	Minimum	Maximum	Mean	SD
CJIF per graduate	0.24	48.56	4.712	5.94
H-index	0	20	3.65	2.95

**Table 5:** Academic characteristics regarding graduates

	N=169	%
University teaching staff		
No	66	40
Yes	103	60
Doctorate grade at graduation		
Good	14	8
Very good	131	78
Excellent	23	14

Determining the variables that impact research outcomes

The linear regression analysis in Table 5 shows that having a higher grade (standardized beta 0.360, CI=0.630-1.376, p<0.001), getting financial help for at least one publication (standardized beta 0.260, CI 0.309-1.016, p<0.001), and working with international partners on at least one publication (standardized beta 0.173, CI=0.114-0.918, p<0.05) were all linked to more publications among graduates. The study found that the cumulative JIF of publications derived from doctoral research was positively associated with higher grades (standardized beta 0.216, CI=1.374-4.268, p<0.001), at least one publication with international collaborators (standardized beta 0.195, CI=1.358-4.077, p<0.001), and a greater number of citations (standardized beta 0.581, CI=0.086-0.128, p<0.001). Earlier graduation was associated with a higher number of citations of WoS publications derived from doctoral research (standardized beta -0.230, CI = -14.29-(-4.25), p < 0.001). Graduates with higher grades at graduation had a higher score of citations (standardized beta 0.489, CI = 25.419-43.819, p < 0.001). Those with at least one financially supported publication (standardized beta 0.159, CI=1.655-18.961, p<0.05) and those who reported at least one international collaboration (standardized beta 0.161, CI=2.274-22.050, p<0.05) also had higher citation scores.

The results of the linear regression analysis indicate that female graduates had a notably higher number of publications upon graduation (standardized beta 0.183, CI = 0.147-1.390, p < 0.05). According to the study, there was a strong link between the number of WoS publications and three things: graduating earlier (standardized beta -0.177, CI = -0.812-(-0.066), p < 0.05); getting a better grade at graduation (standardized beta 0.176, CI = 0.090-1.440, p < 0.05); and existing financial support for at least one publication (standardized beta 0.171, CI = 0.067-1.294, p < 0.05). Participants with higher grades at graduation (standardized beta 0.216, CI=1.040-8.901, p<0.05) and more citations (standardized beta 0.207, CI=0.012-0.123, p<0.05) had a higher cumulative JIF, which is related to the total number of WoS publications at graduation. Furthermore, the results of a linear regression analysis indicate that participants who graduated earlier and with higher grades had a higher number of publications after completing doctoral studies. The standardized beta coefficients were -0.382 (CI = -0.999-(-0.464), p < 0.001) and 0.195 (CI = 0.180-1.130, p < 0.005). Participants who identified as teachers and those who had a higher number of publications at the time of graduation had a higher number of WoS publications after graduation (standardized beta 0.205, CI=0.209-1.055, p<0.005, and respectively standardized beta 0.170, CI=0.023-0.239, p<0.05). Participants with higher grades at graduation (standardized beta 0.216, CI = 1.040-8.901, p<0.05) and more citations (standardized beta 0.207, CI=0.012-0.123, p<0.05) had a higher cumulative JIF, which is related to the total number of WoS publications at graduation. Earlier graduation (standardized beta = -0.142, CI = -0.914-0.119, p < 0.05) and a higher number of citations (standardized beta 0.686, CI = 0.052-0.072, p < 0.001) were associated with a higher H-index among participants (Table 6).

**Table 6:** Simple linear regression analysis An assessment of several factors that may impact the outcomes of research activities among doctoral students. WoS: Web of Science, JIF: Journal Impact Factor, H-index: Hirsch index.

Number of publications derived from doctoral research, indexed in WoS database	Beta		CI		P	
Graduation qualification	0.360		(0.630-1.376)	<0.001		
At least one publication with financial support	0.260		(0.309-1.016)	<0.001		
At least one publication with international collaboration	0.173		(0.114-0.918)	<0.05		
Cumulative JIF (publications derived from doctoral research)	Beta		CI		P	
Graduation qualification	0.216		(1.374-4.268)			<0.001
At least one publication with international collaboration	0.195		(1.358-4.077)			<0.001
Total number of citations (of theses-related publications)	0.581		(0.086-0.128)			<0.001
Total number of citations (publications derived from doctoral research)	Beta		CI			P
Year of doctoral graduation	-0.230		(-14.29- -4.250)			<0.001
Graduation qualification	0.489		(25.419-43.819)			<0.001
At least one publication with financial support	0.159		(1.655-18.961)			<0.05
At least one publication with international collaboration	0.161		(2.274-22.050)			<0.05
Number of all WoS publications at graduation (derived and not derived from doctoral research)	Beta		CI		P	
Gender	0.183		(0.147-1.390)			<0.05
Year of doctoral graduation	-0.177		(-0.812- -0.066)			<0.05
Graduation qualification	0.176		(0.090-1.440)			<0.05
At least one publication with financial support	0.171		(0.067-1.294)			<0.05
Cumulative JIF at graduation (publications derived and not derived from doctoral research)	Beta		CI		P	
Graduation qualification	0.216		(1.040-8.901)			<0.05
Total number of Citations (of theses-related publications)	0.207		(0.012-0.123)			<0.05
Number of WoS publications after graduation	Beta		CI		P	
Year of doctoral graduation	-0.382		(-0.999- -0.464)			<0.001
Graduation qualification	0.195		(0.180-1.130)			<0.005
University teaching staff	0.205		(0.209-1.055)			<0.005
Total number of publications upon graduation	0.170		(0.023-0.239)			<0.05
H-index	Beta	CI			P	
Year of doctoral graduation	-0.142	(-0.914- -0.119)			<0.05	
Total number of citations (publications derived from doctoral research)	0.686	(0.052-0.072)			<0.001	

## Discussion

This observational research offers bibliometric analysis results related to the scientific activity of doctoral graduates from the University of Medicine and Pharmacy in Cluj-Napoca, Romania. The elements of originality refer to evaluating the scientific performance of individual researchers and assessing the productivity and quality of publications resulting from their doctoral research. We accomplish this by analyzing the quantity of publications and the bibliometric indicators of WoS publications from doctoral research, identifying graduates as first authors. The study also monitored the participants' tendencies to publish scientific manuscripts by tracking their publication rates before, during, and after doctoral studies. We also analyzed aspects that could influence doctoral research results.

The results show a notably elevated level of publication activity among doctoral graduates at the University of Medicine and Pharmacy during the 2019-2021 period. Having a substantial number of publications can be beneficial for a doctoral student as it demonstrates their strong research and writing abilities and provides a solid foundation for their future career [29]. Table 1 shows that over a three-year period, 536 articles were published, more than half were WoS-indexed, and almost all of the WoS publications were indexed in journals with JIF. This could be attributed to the 2018 requirements for obtaining the title of doctor, which include meeting the minimum standards as follows: The candidate must have published at least three scientific articles as the first author, sole author, or corresponding author. These manuscripts should include results derived from the content of doctoral research. Doctoral publication requirements vary globally. Several low- and middle-income nations use publishing as a graduation requirement. Pakistan demands one refereed journal paper, Malaysia up to two indexed publications, dependent on the institution, and Iran one to three published or accepted articles before conferring a PhD [30-32]. National or institutional standards in China need one or more peer-reviewed publications in high-impact journals [33]. Most high-income countries (HICs) employ flexible approaches to publishing rather than mandating it. Countries such as the US, UK, and Canada prioritize thesis quality, with publishing recommended but not essential [34]. Article-based PhDs are prevalent in Germany, Finland, Sweden, and the Netherlands, where applicants submit three to five peer-reviewed articles as part of a cumulative thesis without a national mandate, but are well-established in academic practice [35-38]. In Romania, in the period when our study sample performed their PhD studies, it was mandatory to have published/accepted at least one article in a journal indexed in a WoS-rated journal with JIF, and at least two in journals indexed in international databases [24].

Previous research has demonstrated that Romanian graduates produced 260 publications in WoS journals with JIF, more than the 143 from France and 74 from the UK. This was based on a five-year study from 2009 to 2013. The research study did not specify, however, how many of the manuscripts were first-author manuscripts. It also showed that Romania's WoS publishing percentage for overall publications (25%, meaning the percentage of publications indexed in the WoS database and in different international databases) was lower than that of France (89.93%) and the UK (92.5%). In 2018, Romania updated its publication requirements, introducing a set of minimal WoS publications. Before, there were no national legislations in Romania, France, or the UK regarding WoS publications [6]. In a separate comparison, an investigation was conducted to evaluate the efficacy and influence of clinical doctoral theses that originated from the University of Copenhagen. The results indicated a greater rate of publication, with a total of 2534 publications within a five-year period (2013-2017), averaging approximately 500 publications annually [27].

Given the length of time between manuscript submission and publication, it is possible that participants may not have the opportunity to publish all the results of their doctoral studies during their doctoral tenure. Therefore, we wanted to investigate whether individuals who have successfully obtained a PhD degree continue to actively participate in research activities. Our study revealed that post-graduation participants tend to publish, with a total of 154 publications identified and a mean of 0.91 (see Table 1). In comparison, another study collected data on the international publication output of 532 graduates over a five-year period, starting five years after their graduation, using data from PubMed, which resulted in a total of 4,530 articles [39]. In our study, we observed an asymmetry in the publication timeline between graduates from 2019 and 2021, the former having more time to publish their work than the latter, up until the time of our investigation. It might be useful to investigate these aspects using a consistent period, specifically focusing on the number of publications after graduation.

For the quality evaluation, we analyzed publications resulting from their doctoral research, using the same method as for the productivity evaluation. All manuscripts included in our study were peer-reviewed and indexed in the WoS database, indicating high-quality publications. We measured journal quality by the impact factor and the journal rank to assess research performance. As indicated in Table 2, the present study found that most of the WoS publications were indexed in journals with JIF. In our study, the mean JIF was 1.96. However, in a different study that evaluated doctoral theses submitted from 2009 to 2013, Romanian students had a mean impact factor of 1.73 for WoS publications, whereas French and UK students had a higher mean impact factor of 4.42 and 2.88, respectively [6]. Thus, we observed that the impact factor of publications by Romanian graduates tends to increase over time. The assessment of scientific performance strongly correlates with the cumulative JIF associated with the journals that publish manuscripts [20]. We expected this mean value for the cumulative JIF (4.712, Table 4) because most of the publications were in journals with JIF between 1 and 3, and most of the graduates had around 2 WoS publications related to their theses (Tables 1, 2).

We evaluated journal visibility using the JCR ranking system, which classifies journals based on their research domains. Our findings indicate that the proportion of publications in the first quartile (Q1) and second quartile (Q2) was lower compared to the proportion of publications in the third quartile (Q3) and fourth quartile (Q4). The results show that the distribution of journal rankings where graduates publish is consistent with a previous study from 2009 to 2013. Romanian PhD students tend to have a higher proportion of their articles published in Q3 or Q4 journals, while France and the UK exhibit a similar pattern, with a greater proportion of articles published in Q1 or Q2 journals. We attribute the lower proportion of articles included in the present research that were published in Q1 or Q2 journals to the high article processing charges (APCs) typically associated with top-ranked open-access journals. The doctoral theses analyzed in this study, defended between 2019 and 2021, correspond to doctoral research initiated between 2015 and 2017, a period during which Romania was classified by the World Bank as an upper-middle-income country [40].

About 10 years ago, Urda-Cimpean et al. drew attention to the fact that researchers in low- and middle-income countries faced relatively high costs for accessing open-access journals [6]. This could potentially obstruct the publication of research findings and hinder the career progression of these researchers [41]. Article Processing Charges (APCs) for open-access publication are often high, especially for journals classified in the first and second quartiles (Q1 and Q2), hence limiting accessibility for researchers from low- and middle-income countries (LMICs). Jahn and Tullney (2016) emphasize that cost is the predominant obstacle to open access publishing, disproportionately impacting researchers in underfunded areas and fields [42]. International guidelines advocating for transparency, fair APC waiver rules, and the establishment of alternative models such as diamond open access, which is accessible at no cost to both users and writers, may provide sustainable alternatives for academics in LMICs [43].

However, the concern of funding for open-access publishing of publications deriving from research theses is prevalent even in countries with high incomes. To cover article-processing costs, authors sometimes rely on financing from grant organizations or academic institutions. The payment of these costs is split across the budgets of grant agencies, academic institutions, and libraries, or is financed by personal budgets. A previous study revealed that Germany, Austria, and the UK have different open-access publication financing strategies. The Deutsche Forschungsgemeinschaft (DFG) funds article processing costs (APCs) in Germany through a centralized scheme established in 2011 with rigorous requirements, including a €2,000 price ceiling and the exclusion of hybrid journals. Since 99% of publications were published in completely open-access journals, this approach has clearly favored them. In contrast, the Austrian Science Fund (FWF) funds both completely open-access and hybrid periodicals. FWF spent most of its 2014-2015 budget on hybrid journals with higher APCs. The Wellcome Trust and Jisc finance completely open-access and hybrid publications in the UK. UK statistics show hybrid journals get the most funding, with APCs typically outperforming completely open-access venues. This difference shows how national funding regulations and eligibility requirements affect institutional publishing and open-access spending [42].

Similarly, in Romania, the Iuliu Hațieganu University of Medicine and Pharmacy has also introduced in the last few years a funding program to partially reimburse APCs for open-access articles published in Q1 and Q2 journals. This reimbursement varies between €1,000 and €2,000 and is drawn from institutional funds, prioritizing support for articles where the university-affiliated researcher is listed as the first author [44]. In 2024, the World Bank classed Romania as a high-income nation [40]. This transition offers a significant opportunity for future studies to investigate whether this economic advancement will be mirrored in the scientific output of Romanian PhD students.

Another possible explanation is related to the low funding for research, limited possibilities to access national funds for research projects, including for PhD students as well as their coordinators, which impacts the research capacity, the quality and innovation of results. According to Eurostat, in 2023, Romania was among the five countries in the European Union that reported research and development expenditure that was below 1% of their GDP. The countries with the lowest investment in research were those that had joined the EU in 2004 or more recently, Romania, Malta, Cyprus, and Bulgaria [45]. 

Clarivate Analytics, Elsevier, and Google Scholar are the primary entities in the scholarly publishing industry that gather data on article citations. Measures of author and journal influence, which depend on article citations, utilize one of these sources and might show slight variations due to differences in journal coverage [46]. Our research findings indicate that 298 (80%) of these WoS-related publications received at least one citation. However, over half of these publications received between one and 10 citations. Most graduates received at least one citation, with a mean of 18.15 citations per graduate (Tables 2, 3). The Copenhagen study conducted from 2013 to 2017 showed a mean annual citation rate of 3.9 times for the published manuscripts over the five-year period [27].

According to Sahel et al., assessing the performance of individual scientists is crucial in research evaluation. The results of these assessments have significant implications for institutional research strategies, such as funding, recruitment, termination, and promotions [47]. Citation-based measures frequently quantify the impact of individual scientists. The H-index is the most frequently used measure in this context. Our findings show that the graduates had an H-index mean of 3.65, with a range of 0 to 20 (see Table 4). A study conducted in Hungary found that the H-index score of medical doctoral graduates was 3.32 [48].

The traditional format of a doctoral thesis, characterized by a single author and monograph-style layout, has experienced a decline in prominence within the fields of natural and biomedical sciences. In its place, a contemporary approach to doctoral theses has emerged, wherein a compilation of manuscripts and publications authored by multiple individuals has largely taken precedence. Researchers recognize that the number of authors involved in a paper significantly influences the number of citations a paper receives across various academic fields. Incorporating multiple authors in a study generally leads to higher-quality research due to the increased effort and expertise contributed to the work. Increased authorship leads to greater awareness of authors, dissemination of their publications, and potential citation in other scholarly works [49]. Hagen proposes a harmonic counting method that determines authorship credit fairly and transparently. In their study, he suggests that all coauthors should share the publication credit, with the first author receiving the most credit. As the number of authors increases, the credit per author decreases [50-52].

In the present investigation, graduates have a mean of 5.6 co-authors. Notably, graduate students represent the first authorship of all the publications examined in this study. By examining the mean number of co-authors reported in the comparative study, it is evident that Romanian graduates have a mean of 4.54 co-authors, while UK graduates have a mean of 4.96 co-authors. Additionally, French graduates have a mean of 7.67 co-authors, and a study on doctoral theses of Copenhagen graduates reveals a mean of 7.4 co-authors [6,27]. These findings are consistent with the data from the analyzed studies, with no significant discrepancies observed.

Over the past decade, there has been an increasing focus on the development of collaborative, transdisciplinary scientific teams to facilitate the advancement of knowledge creation and dissemination. The current study found that almost all graduates had participated in interdisciplinary teams, with the majority of publications reporting on this type of collaboration. Interdisciplinary research collaboration is essential for conducting transformative scientific research and accelerating innovation [53]. In a study that examined collaboration patterns at a single academic medical center, the authors observed statistically significant associations between specific authorship patterns and publication in high-impact journals. There was a significant correlation between the presence of professors, research scientists, and basic science researchers as authors and publication in high-impact journals. Researchers found an association between academic rank mixing and publication in high-impact journals [54].

Thelwall et al. provided direct evidence of the superior quality of journal articles authored by international teams compared to those conducted by national teams [55]. Their findings are primarily applicable to the UK, but they are also likely to hold true for other countries that produce high-impact research and may be relevant to most advanced economies. In our study, we found that the rate of international collaboration was lower than that of interdisciplinary collaborations (Table 2). However, Thelwall et al. highlight that international collaboration may be harmful in some fields and ineffective in others. In this context, it seems prudent to be careful in selecting fields for international research funding and to allow national research to grow in areas where it excels [55].

In their study, Urda-Cimpean et al. found that over half of doctoral theses in France and the UK receive financial support from research projects. However, the situation in Romania has been unclear, likely due to the lack of regulations requiring the acknowledgment of financial support in PhD theses [6]. Recently, there have been changes in research grants for doctoral students. Currently, our university offers a doctoral research intern grant, which is the most accessible option. Students are now required to acknowledge any financial support they receive in their work. Table 2 reveals that approximately half of the publications received support from scientific grants, and Table 3 reports that half of the graduates received financial support. Most of these graduates obtained doctoral research grants from the University of Medicine and Pharmacy Iuliu Hatieganu. 

In our study, we have identified several hypotheses that could potentially impact the publication of research findings. Upon graduation, female graduates published a higher number of publications, suggesting a greater inclination to publish manuscripts not derived from their doctoral research. Although the gender of the graduates didn't influence the number of publications derived from their doctoral research (see Table 6). However, we could also attribute these findings to the significantly higher percentage of female authors in Romania. Urda-Cimpean et al. corroborate this finding, as their study reveals a higher female graduation rate, which aligns with the proportion of women in our own study [6].

Graduates with higher qualifications at graduation demonstrated a higher quantity of both publications derived and not derived from doctoral research. Furthermore, they demonstrated a tendency to continue their involvement in publishing activities after graduation. Furthermore, graduates who achieved a higher qualification at graduation demonstrated a greater impact in their publications, as evidenced by a higher number of citations. These graduates also tended to publish articles related to their theses as well as other research in prestigious journals prior to graduation. Their higher cumulative JIF (see Table 6) supports this. As expected, graduates with more citations of publications derived from doctoral research had a higher H-index (see Table 5). 

Graduates who completed their studies earlier had higher citation counts for thesis-related publications. Also, postgraduates demonstrated a higher publication rate and, as expected, had a greater H-index when we performed this investigation (see Table 6). These features likely stem from the graduates having more time than those who graduated later to dedicate themselves to publishing and obtaining references after graduation. Most citations typically occur several years after a manuscript's publication. Consequently, for future investigations, it may be appropriate to analyze the citation rate within specific time frames for each year following graduation.

Furthermore, graduates who received a higher number of citations tended to publish in high-quality journals. This is because both the cumulative JIF of WoS publications resulting from doctoral research and the cumulative JIF at graduation (which also includes the JIF of publications unrelated to the doctoral thesis) were higher (see Table 6). Despite ongoing debates, extensive research consistently supports the notion that articles published in high-impact journals receive more citations [56]. This is primarily due to the perception that these journals contain superior content and naturally attract greater attention [57].

Graduates who reported receiving financial support for their doctoral research tended to publish a greater number of articles based on their research findings (see Table 6). This, in turn, had a positive influence on the impact and visibility of their work, resulting in a higher number of citations for publications related to their theses. Furthermore, our findings suggest that funded graduates were able to publish additional manuscripts unrelated to their doctoral research prior to graduation (see Table 6). These findings demonstrate that research funding is crucial for medical research.

Graduates who had international collaborators during their doctoral work tended to publish more results from their research. Additionally, these graduates had a greater impact and visibility, as evidenced by a higher cumulative impact factor and a greater number of citations for their theses-related publications (see Table 6). According to Wagner et al., international collaborations tend to have a greater influence on citation impact in most subject areas, likely due to the greater collaborative network they offer [58]. According to Table 6, the graduates who reported employment as university teaching staff continued to publish after graduation. In their study, Pfeiffer et al. noted that the increased publication productivity, particularly after graduation, and the significantly higher rate of original article publications among these graduates suggest a greater likelihood of pursuing academic medicine and potentially an academic career [59].

Strengths and limitations of this study

This study provides valuable insights into the productivity and quality of publications resulting from doctoral research at one of Eastern Europe's most prestigious health-care universities. The study population, the thesis-related manuscripts, and the scientometric indicators of the publishing journal were comprehensive and contained no missing information. For this study, we utilized diverse and reliable sources, such as doctoral theses, the Web of Science database, and the curriculum vitae of participants (available on the university website) to ensure data validity. The primary constraint is to monitor post-graduation publications. Authors often face challenges in assessing their impact due to duplicate names caused by inconsistencies in the name they use (e.g., Jackson JL vs. Jackson J) or name changes [46]. Additionally, there is an asymmetry in the publication time frame between graduates from 2019 and those from 2021, with the former having a longer period to publish their work in comparison to the latter. Future research should investigate these aspects within a comparable timeframe in terms of the number of publications post-graduation.

## Conclusions

The findings indicate that the doctoral theses conducted at Iuliu Hatieganu University of Medicine and Pharmacy provided a high scientific output. Most WoS manuscripts are peer-reviewed, with many appearing in top-ranked journals. Our study revealed a clear relationship between the productivity, influence, and visibility of the graduates' publications and the grade received at graduation. Many graduates continue to produce new research after graduation, demonstrating engagement with scientific work and publishing beyond dissertation projects.

The study findings validated that both financial assistance and collaboration with international researchers had a significant impact on the visibility and quantity of publications. Thus, this research offers new quantitative and qualitative evidence that can contribute to discussions on academic performance assessments and mentoring PhD students. Our findings highlight the factors that can influence research outcomes. Nevertheless, their generalizability is most reliable within comparable academic and institutional settings. Although, whether medical research doctoral candidates finish their doctoral program with appropriate scientific progress is not exactly known. Future research could further investigate the opinions and experiences of doctoral students regarding their research, as well as factors that impact research outcomes.
